# A Concurrent Finding of Pulmonary Sarcoidosis, Primary Hyperparathyroidism, Thyroid Nodules, and Adrenal Tumor in a Sexagenarian

**DOI:** 10.7759/cureus.100549

**Published:** 2026-01-01

**Authors:** Alamin Alkundi, Rabiu Momoh

**Affiliations:** 1 Diabetes and Endocrinology, William Harvey Hospital, Ashford, GBR; 2 Critical Care, Medway Maritime Hospital, Gillingham, GBR

**Keywords:** adrenal disorder, calcium metabolism, conn's syndrome, endocrinology, multiple endocrine neoplasia syndrome, primary hyperparathyroidism, rare associations, sarcoidosis, syndromes, thyroid nodules

## Abstract

The concurrent presence of pulmonary sarcoidosis, primary hyperparathyroidism (PHPT), thyroid nodules, and adrenal tumor in an individual or patient is rare and complex. We present a rare scenario of the presence of the above conditions in a 60-year-old hypertensive patient (with a high plasma aldosterone-to-renin ratio) who was followed up in an endocrinology clinic at a district general hospital. We have reviewed the literature in a bid to offer insights into possible pathophysiological connections and underlying mechanisms to account for this.

## Introduction

A concurrent finding of pulmonary sarcoidosis, primary hyperparathyroidism (PHPT), thyroid nodules, and an adrenal tumor in a single patient represents a rare and diagnostically complex clinical scenario. Sarcoidosis, a multisystem granulomatous disease, is known to cause hypercalcemia through an increased production of 1,25-dihydroxyvitamin D by activated macrophages. Its coexistence with PHPT - another condition also characterized by hypercalcemia - poses a unique diagnostic challenge. Literature suggests that this dual pathology occurs more frequently than chance alone would predict, with over 30 cases reported since 1958, highlighting the importance of considering sarcoidosis in patients with persistent hypercalcemia post-parathyroidectomy [1].

Adding to the complexity, the presence of thyroid nodules and an adrenal tumor raises the possibility of underlying endocrine neoplasia syndromes (e.g., multiple endocrine neoplasia (MEN) syndromes - MEN 1, MEN 2A, MEN 2B, MEN 4) or genetic predispositions [2]. Thyroid nodules are common in the general population. Their coexistence with adrenal tumors may portend a significant endocrine dysregulation [3]. In this case, the constellation of these findings in a sexagenarian suggests consideration of both sporadic and syndromic aetiologies, and emphasizes the importance of comprehensive imaging, biochemical evaluation, and genetic screening towards uncovering the underlying pathology. This case report aims to contribute to the growing body of literature on complex endocrine and granulomatous disease intersections and to offer insights into diagnostic strategies and therapeutic decision-making. 

## Case presentation

**Figure 1:**
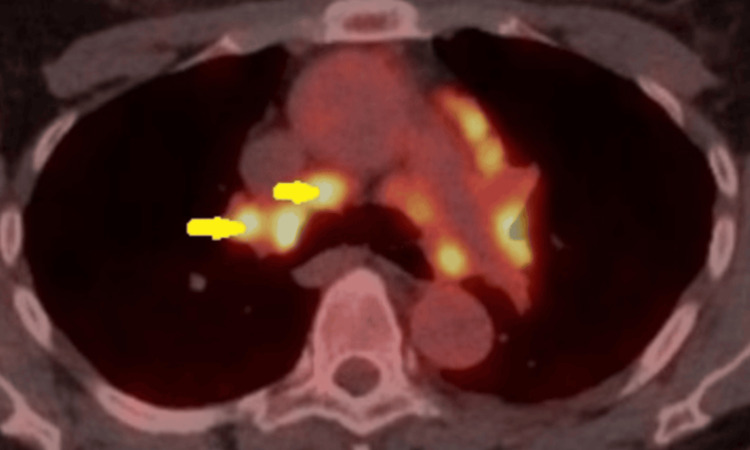
Transverse section of a PET CT study revealing fluorodeoxyglucose (FDG)-avid mediastinal and bilateral hilar lymph nodes PET CT: Positron Emission Tomography Computed Tomography.

We report the case of a 60-year-old asymptomatic female patient who was reviewed at an endocrinology clinic following a referral for clinical conditions and radiological findings that required ongoing endocrinological input. She was noted on her records to have a longstanding assessment with pulmonary sarcoidosis, which was confirmed on an endoscopic ultrasound transbronchial needle aspiration of her mediastinal lymph nodes. Respiratory physicians were managing her in an outpatient clinic. Figure 1 depicts the Positron Emission Tomography Computed Tomography (PET CT) scan showing multiple mediastinal lymph nodes in the patient.

She had a right parathyroid adenoma excised about six years prior and underwent a left hemithyroidectomy (for a multinodular goiter and multiple U2 nodules affecting mainly the inferior thyroid lobe) at the same surgery. Her records revealed she had osteoporosis affecting her spine and both forearms.

She reported a positive family history of parathyroidectomy in five family members and reported a first-degree relative who had pancreatic cancer. The genetic screen study for MEN syndrome was negative. An incidental finding of a small left adrenal focal lesion (likely adenoma) was noted on a CT of the abdomen and pelvis, done as part of adjoining investigations, six years before her parathyroidectomy.

Her comorbidity also included hypertension, for which an evaluation revealed the presence of a high aldosterone-to-renin ratio (5750 pmol/L per µg/L/hr (reference value: < 651 pmol/L per µg/L/hr)), highly suggestive of Conn's syndrome. She was found with a left adrenal nodule that was not radiotracer 18F-fluorodeoxyglucose (FDG) avid. A 24-hour urinary sample collection to measure cortisol and metanephrines was negative. Her blood pressure measurements were controlled on daily oral amlodipine and lisinopril.

**Figure 2:**
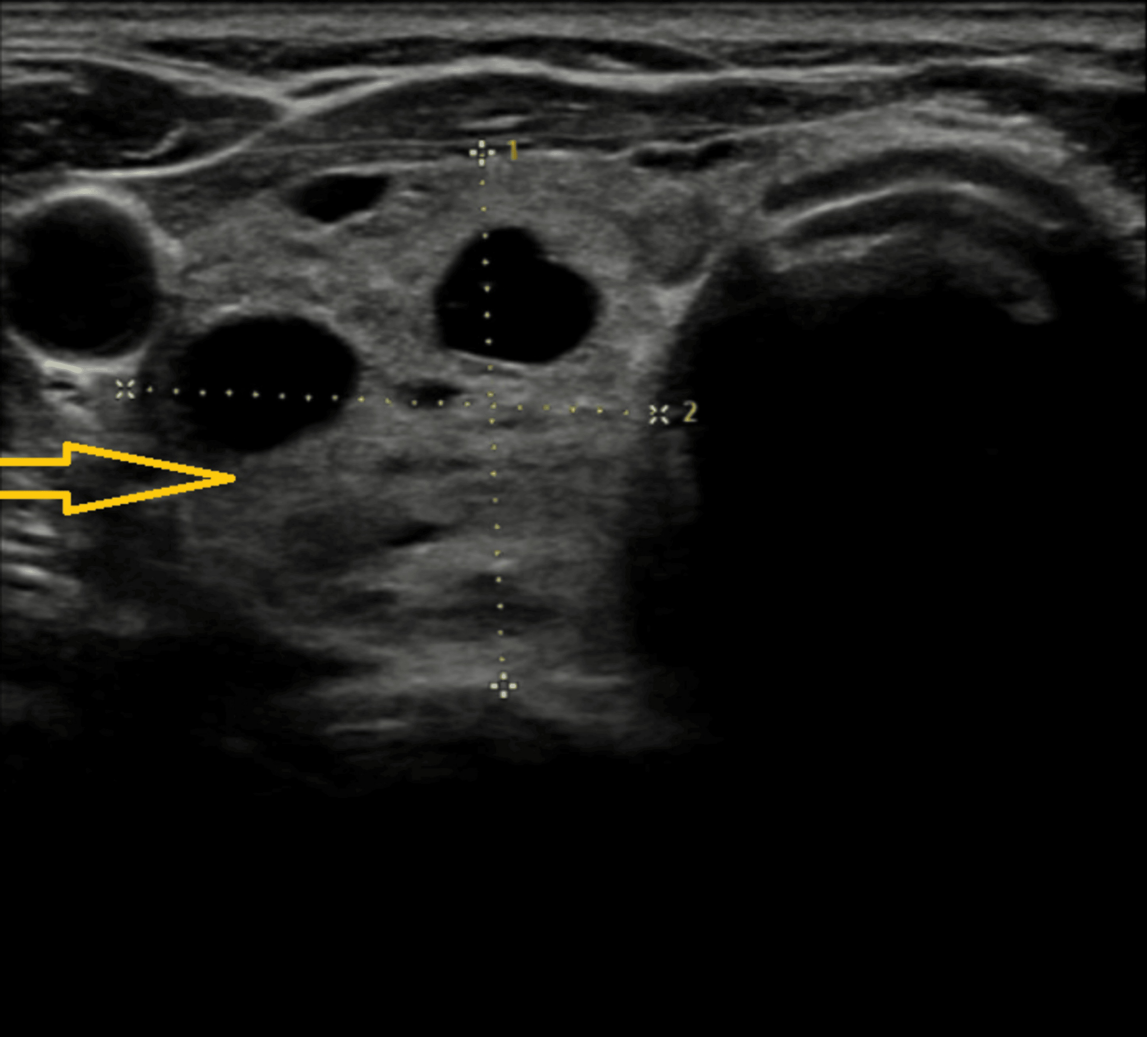
Transverse neck ultrasound view section revealing multiple U2 nodules in the right thyroid lobe

A recent thyroid ultrasound scan study of her neck revealed that her left thyroid bed appeared clear, suggesting evidence of a previous left hemithyroidectomy. The right thyroid lobe appeared mildly enlarged with multiple nodules, predominantly cystic (largest measuring 14 x 15mm), a few spongiform nodules (largest measuring 19 x 11 mm), and isoechoic nodules (largest measuring 8 x 8 mm) (Figure 2).

Normal appearance of the parotids and submandibular glands was noted. No significant lymphadenopathy in the lateral neck was found.

She underwent a saline infusion test to further evaluate her primary hyperaldosteronism for the probable consideration of surgery. The saline infusion test, however, was not confirmatory for primary hyperaldosteronism (Table 1). 

**Table 1:** Outcome of the patient's saline infusion test

Time (minutes)	Plasma aldosterone (pmol/L)	Plasma renin concentration (mIU/L)
0	634 (ref: 55–250 pmol/L - supine)	2.8 (ref: <10 mIU/L (supine))
4 hours post-intravenous saline administration	<108 (ref: Typically less than 140 pmol/L)	2.1 (ref: <10 mIU/L (supine))

## Discussion

We have presented the findings of the concurrent occurrence of assessments of pulmonary sarcoidosis, PHPT, thyroid nodules, adrenal tumor, hyperaldosteronism, and osteoporosis in a sexagenarian with a positive family history of parathyroidectomies among five family members. Multi-system endocrine and granulomatous disorders, as noted in our case, often present diagnostic and therapeutic challenges due to overlapping biochemical and clinical features. We have reviewed the pathophysiological intersections, diagnostic dilemmas, and genetic implications of these coexisting conditions.

Sarcoidosis is a granulomatous disease characterized by non-caseating granulomas, commonly affecting the lungs. It is known to cause hypercalcemia due to increased 1α-hydroxylase activity in macrophages, leading to elevated levels of active vitamin D (calcitriol) [4]. Interestingly, sarcoidosis can coexist with PHPT, complicating the interpretation of a hypercalcemia finding. The index case under review, however, was not complicated by significant hypercalcemia. She was otherwise noted with osteoporosis affecting the spine and forearms. Schweitzer et al. described cases where sarcoidosis and PHPT occurred synchronously, suggesting a non-random association [1].

PHPT is most often caused by parathyroid adenomas but may also arise in familial syndromes such as MEN types 1 and 2A, and hyperparathyroidism-jaw tumor syndrome. Familial isolated PHPT (FIHP) is another variant, often presenting earlier and with multiglandular involvement [5]. The presence of parathyroidectomies in five family members in this case suggests a heritable form. The genetic screen for MEN syndromes undertaken on the patient was negative.

Thyroid nodules are common in the general population, but their presence in patients with MEN syndromes or other endocrine disorders may suggest a syndromic association. Studies have shown increased prevalence of thyroid nodules in patients with adrenal incidentalomas, possibly due to shared endocrine dysregulation [5]. In our case, the thyroid U2 nodules identified may represent benign hyperplasia or be part of a broader unknown endocrine neoplastic syndrome.

Primary aldosteronism, often caused by adrenal adenomas, leads to hypertension and hypokalemia. Recent literature has linked it to secondary hyperparathyroidism due to aldosterone-induced urinary calcium loss and subsequent parathyroid hormone elevation [6]. Ceccoli et al. demonstrated that primary hyperaldosteronism patients had significantly higher parathyroid hormone levels and reduced bone mineral density (BMD), which improved post-treatment [7]. In our case, our patient had a high plasma aldosterone-to-renin ratio and had hypertension as a comorbidity. A left adrenal nodule (non-FDG avid) was also found on radiologic assessment. She also had a negative saline infusion test. Akkuş et al. (2019) suggested that FDG activity could be variable in functional adrenal adenomas [8]. Also, the saline infusion test has a false negative rate of 30% in determining primary aldosteronism [9].

Osteoporosis in our patient may be multifactorial - stemming from her hyperaldosteronism as noted above, her primary hyperparathyroidism, sarcoidosis, or a combination of the aforementioned factors. PHPT increases bone turnover, particularly affecting cortical bone, while primary aldosteronism contributes to bone loss via secondary hyperparathyroidism. Sarcoidosis may also impair bone health through chronic inflammation and steroid use. Salcuni et al. emphasized the need to consider primary aldosteronism as a secondary cause of osteoporosis, especially in hypertensive patients [10].

The clustering of endocrine tumors and nodules in this patient raised suspicion for MEN syndromes, which was, however, ruled out based on genetic studies done on the patient. MEN1 typically involves parathyroid, pancreatic, and pituitary tumors, while MEN2A includes medullary thyroid carcinoma, pheochromocytoma, and PHPT. Cetani et al. recommend early genetic evaluation in patients with familial PHPT to guide surgical and surveillance strategies [5].

Differentiating between sarcoidosis-induced hypercalcemia and PHPT is critical, as management strategies differ. Sarcoidosis responds to corticosteroids, while PHPT requires surgical intervention [11]. Lief et al (1969) suggested considering a diagnosis of PHPT in a sarcoidosis patient with hypercalcemia that is accompanied by a finding of hypophosphatemia and resistance to steroid therapy [12]. Our patient had ongoing surveillance management for her pulmonary sarcoidosis, but had an excision surgery to treat her parathyroid adenoma. There were no hypercalcemia readings in our patient despite these diagnoses.

The management of this index complex endocrine case required a multidisciplinary approach. Parathyroidectomy was undertaken for her PHPT. A possible adrenalectomy option was being considered for the patient who had required a mineralocorticoid receptor antagonist in the treatment of her hypertension and hyperaldosteronism till the time of this report. Sarcoidosis may require corticosteroids, and osteoporosis management includes the use of bisphosphonates or denosumab. Tailoring therapy to address each condition while considering interactions is vital. Family screening and genetic counselling were recommended to her and her blood-related family members. The Oxford University Hospitals, UK, recommend genetic panel testing for suspected cases of familial PHPT, and this testing panel include testing for mutations on MEN1, cell division cycle 73 (CDC73), and cyclin-dependent kinase inhibitor 1B genes (CDKN1B) [13].

## Conclusions

This case exemplifies the intricate interplay between granulomatous disease, endocrine tumors, and metabolic bone disorders. The coexistence of sarcoidosis, PHPT, thyroid nodules, adrenal tumor, osteoporosis, and a familial endocrinopathy predisposition underscores the importance of comprehensive evaluation of patients. Literature supports the need for genetic testing, multidisciplinary management, and vigilant follow-up in such patients. The patient under review was normocalcemic despite having risk factors for hypercalcemia. A saline infusion test was not confirmatory for the presence of primary hyperaldosteronism in this hypertensive patient despite the presence of a raised baseline plasma aldosterone level and a high plasma aldosterone-to-renin ratio. Further research activities are suggested based on the findings and associations in this case report. 
